# Real‐Time In Situ Imaging of Aggregation‐Induced Emission and Solvent‐Guided Morphogenesis of a “V‐Shaped” 4‐Amino‐1,8‐Naphthalimide Tröger's Base Supramolecular Scaffold

**DOI:** 10.1002/smsc.202500386

**Published:** 2025-10-21

**Authors:** Sankarasekaran Shanmugaraju, Deivasigamani Umadevi, Aramballi J. Savyasachi, Chris S. Hawes, Jonathan A. Kitchen, Gavin J. McManus, Thorfinnur Gunnlaugsson

**Affiliations:** ^1^ School of Chemistry and Trinity Biomedical Sciences Institute (TBSI) Trinity College Dublin The University of Dublin Dublin 2 Ireland; ^2^ Department of Chemistry Indian Institute of Technology Palakkad Palakkad Kerala 678623 India; ^3^ School of Chemical and Physical Sciences Keele University Keele ST5 5BG UK; ^4^ Chemistry, School of Science Auckland University of Technology Auckland 0632 New Zealand; ^5^ School of Biochemistry and Immunology Trinity Biomedical Sciences Institute (TBSI) Trinity College Dublin The University of Dublin Dublin 2 Ireland

**Keywords:** 1,8‐naphthalimide, aggregation‐induced emission, real‐time visualization, self‐assembly, solvatochromism, solvent‐guided morphogenesis, Tröger's base

## Abstract

The influence of solvent polarity on the self‐assembly processes and its effect on the morphological outcome of self‐assembled aggregates is another domain that requires a comprehensive study. The present investigation aims to address these issues by employing a unique “V‐shaped” luminogen (**TBNap**, N‐(3‐pyridyl)‐4‐amino‐1,8‐naphthalimide Tröger's base), where the two 1,8‐naphthalimide units are nearly orthogonal to each other. The **TBNap** is synthesized in high yield and fully characterized using standard characterization methods, including X‐ray diffraction analysis, which reveals distinctly different structural arrangements of **TBNap** crystallized as different solvates in various solvent media. Furthermore, due to its internal charge transfer nature, the **TBNap** exhibits positive solvatochromism and solvent‐guided morphogenesis. Given the unique structure, **TBNap** displays aggregation‐induced emission enhancement in THF‐H_2_O medium and forms self‐assembled fluorescent nanoaggregates as imaged using different microscopic imaging techniques such as scanning electron microscopy (SEM) and confocal fluorescence microscopy. Furthermore, the latter is employed to demonstrate the in situ real‐time visualization of these fluorescent nanoaggregates formations in native conditions and correlate the morphological outcome with SEM imaging.

## Graphical abstract

It is demonstrated that the self‐assembly process of the 3‐pyridyl‐based **TBNap** structure is highly solvent deepened. X‐ray diffraction analysis confirms that the **TBNap** structure possesses solvent‐depended packing arrangements in the crystalized form. This packing is translated into aggregation‐induced emission enhancement where fluorescent nanoaggregates are formed in THF‐H_2_O solution as imaged in real‐time using confocal fluorescence microscopy.

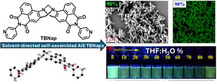

## 1 Introduction

One of the preeminent challenges of supramolecular and material chemistries is to construct more complex structures and higher‐order materials through bottom‐up self‐assembly processes.^[^ ^1^, ^2^ ^]^ While such approaches are beautiful, the fundamental problem is the complexity and the unpredictability of the supramolecular processes (i.e., a wide range of noncovalent interactions) involved.^[^ ^3^ ^]^ Consequently, it is more common that such endeavors translate to unforeseen or “serendipitous” discoveries rather than the expected and carefully planned ones! Despite this, supramolecular self‐assembly has become a powerful synthetic strategy employed to engineer and create large complex structures and functional hierarchical materials that possess properties different from the starting building units.^[^ ^4^, ^5^ ^]^ Such work is often inspired by the fact that precise self‐assembly pathways in living systems create complex biomolecules.^[^ ^6^, ^7^ ^]^

The spontaneous supramolecular self‐assembly or self‐aggregation of chemical entities is a fundamental process and an attractive strategy for generating highly ordered structures and responsive functional materials. However, the precise mechanistic pathways by which the chemical entities are transformed into supramolecular aggregates, real‐time visualization of aggregate formations, and control of assembly processes at the molecular level are still relatively opaque. The self‐assembly processes rely on the information encoded in the predesigned building units.^[^ ^2^, ^4^ ^]^ In the self‐assembly process, the discrete chemical entities spontaneously organize into highly ordered architectures driven by dynamic reversible chemical interactions based on encoded information.^[^ ^8^, ^9^ ^]^ The formation of desired supramolecular architectures is thermodynamically controlled, in which the appropriate complementary building units assemble and disassemble until stable, error‐corrected molecular ensembles are formed.^[^ ^10^, ^11^ ^]^ Previously, it was believed that ordered structures formed by the self‐assembly process were merely controlled by thermodynamic means.^[^ ^12^, ^13^ ^]^ However, recent studies suggest that the formation of ordered supramolecular structures is often the outcome of both thermodynamic and kinetic‐controlled processes within complex equilibrium conditions.^[^ ^14^, ^15^ ^]^

It is commonly accepted that control over self‐assembly processes at the molecular level influences the macroscopic level outcome and facilitates tuning of the bulk material properties.^[^ ^16^, ^17^, ^18^, ^19^ ^]^ In a given solution, the self‐assembly process involves multiple mechanistic pathways leading to the generation of ordered supramolecular structures.^[^ ^18^ ^]^ However, the precise control of such complex supramolecular pathways and managing various factors that influence the self‐assembly process in solution is challenging to achieve.^[^ ^20^ ^]^ Second, in situ, direct visualization of self‐aggregation of discrete molecular entities into supramolecular structures having unique morphological features in solution is difficult and is in high demand in supramolecular activities.^[^ ^1^, ^21^, ^22^ ^]^ In light of this, De Cola et al. have studied the complex self‐assembly pathways involved in the interconversion of a simple Pt(II) coordination complex into morphologically distinct molecular aggregates having different emission properties and elucidated the self‐assembly pathways in real‐time by monitoring the fluorescence images of each of the aggregate formation.^[^ ^23^ ^]^ In another study, Tang and coworkers demonstrated the direct visualization of the chitosan‐based gelation process using the prototypical aggregation‐induced emission (AIE) fluorogenic tetra‐phenylethylene and proposed the precise mechanism for the hydrogel formation.^[^ ^24^ ^]^ However, the real‐time in situ visual imaging of the AIE process of finite chemical entities in a binary solvent system and understanding of the morphological features of aggregates formed from AIE processes at the molecular level is a relatively unexplored area of research. Therefore, a suitable method for the direct visual imaging of AIE aggregate formation will enable elucidation and control of the mechanistic pathways of the self‐aggregation processes.^[^ ^25^, ^26^ ^]^ Further, the influence of solvent polarity on the self‐assembly processes and its effect on the morphological outcome of self‐assembled aggregates is another domain that requires a comprehensive study.^[^ ^27^, ^28^ ^]^

With this in mind, we set out to carry out the present study. We address these issues using a “V‐shaped” luminogen (**TBNap**, *N*‐(3‐pyridyl)‐4‐amino‐1,8‐naphthalimide Tröger's base). **TBNap** exhibits aggregation‐induced emission enhancement (AIEE) property in the THF‐H_2_O solvent system and solvent‐guided micro‐ and nanostructures possessing remarkably distinct morphology, which was probed employing different microscopy techniques. The orthogonal Tröger's base (**TB**) motif has much to offer when it comes to developing novel functional structures, and its real value is not even close to being fully realized. With **TBNaps**, we are ideally positioned to explore their use as versatile scaffolds and realize their vast untapped potential within supramolecular structures and materials. The **TBNap** is a relatively new and unexplored building unit with readily adaptable and modular features for targeting specific structures and applications that possess an angular structure. Hence, the orthogonal geometry, with two points of connectivity, can self‐assemble through dipole‐dipole and π−π stacking interactions. Moreover, they are inherently chiral due to the presence of two stereogenic nitrogen atoms situated within the 1,5‐diazocine ring,^[^ ^29^, ^30^ ^]^ which gives rise to a *C*_2_ axis of symmetry and places the two naphthalimide units close to orthogonal to each other with a small “*helical twist”*.

**TBNap** is highly soluble in THF and displays a moderate emission in THF solution. However, adding H_2_O (defined as a poor solvent) induces enhanced fluorescence emission and results in fluorescent aggregate formation. Taking the advantages of intrinsic fluorescence emission of **TBNap**, we used confocal fluorescence microscopy to directly visualize the AIEE processes in real‐time and imaged concomitant morphological changes of self‐assembled aggregates in binary solution. To the best of our knowledge, reports on in situ real‐time visualization of the AIEE process of a luminogen and morphological properties are rare. Also, the demonstration of solvent‐guided morphogenesis of a fluorophore in different polar solvents is unprecedented. Below, we provide a full account of our investigation, which includes a detailed discussion of the synthesis, photophysics, solvent‐guided morphogenesis, and real‐time visualization of the AIEE process, including the morphological outcome of aggregates emanating from the AIEE process of a new **TBNap** luminogen.

## 2 Results and Discussion

### 2.1 Synthesis, Structural Characterization, and Solvatochromism of **TBNap**

**Scheme 1:**
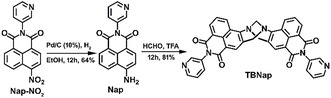
The synthesis of **TBNap** from its nitro‐precursor **Nap‐NO**_**2**_.

As shown in **Scheme** **1**, 4‐amino‐1,8‐naphthalimide derived di‐3‐pyridyl‐Tröger's base (**TBNap**; *Bis*‐[*N*‐(3‐pyridyl)]‐9,18‐methano‐1,8‐naphthalimide‐[*b,f*][1,5]diazocine) was obtained as a racemic mixture in 81% yield from the precursor, *N*‐(3‐pyridyl)‐4‐amino‐1,8‐naphthalimide (**Nap**), which was freshly obtained from *N*‐(3‐pyridyl)‐4‐nitro‐1,8‐naphthalimide (**Nap‐NO**_**2**_) after Pd/C‐based reduction reaction (for details see Experimental Section).^[^ ^31^, ^32^ ^]^ The structure was used in its racemic form in the current study. **TBNap** was found to be soluble in most common organic solvents and fully characterized using standard spectroscopic techniques such as Fourier transform infrared (FTIR), nuclear magnetic resonance (NMR) (^1^H and ^13^C), and high‐resolution mass spectrometry (HRMS) (for full spectroscopic characterization of **Nap‐NO**_**2**_, **Nap**, and **TBNap** see Supporting Information).

The FTIR spectrum showed a sharp band at 1245 cm^−1^ corresponding to the C–N stretching vibration of Tröger's base moiety (Figure S2, Supporting Information). The complete disappearance of the NH_2_ stretching bands at 3327 cm^−1^ of **Nap** (See Figure S1, Supporting Information) confirms the formation of the diazocine ring of the Tröger's base unit. The C = O stretching vibrations of the imide moiety of **TBNap** were observed as an intense peak at 1703 cm^−1^ (See Figure S2, Supporting Information). The ^1^H NMR spectrum of **TBNap** confirmed its identity by the presence of two well‐separated doublets between 5.24 and 4.69 ppm and a singlet at 4.79 ppm, corresponding to the methylene (–CH_2_N–) protons of the diazocine (TB) moiety, while also reflecting the *C*_2_ symmetry of the molecule (See Figure S5, Supporting Information).^[^ ^33^ ^]^ The aromatic proton resonances appeared at 8.62–7.54 ppm corresponding to 3‐pyridyl and naphthalene moieties. The HRMS analysis showed a sharp peak at *m/z* = 615.1770 corresponding to the molecular ion [M + H]^+^ of **TBNap** (See Figure S8, Supporting Information). The molecular structure of **TBNap** was further confirmed by single‐crystal X‐ray diffraction analysis of suitable crystals obtained from different solvents, which demonstrated the effect that the solvent has on directing the assembly formation for such orthogonal systems possessing internal charge transfer (ICT) character. Single‐crystals of **TBNap** as the THF, DMSO, or CH_2_Cl_2_ solvates were obtained by slowly evaporating a concentrated solution at ambient temperature, respectively (Data collection and refinement parameters are summarized in Table S1, Supporting Information).

The neutral solvates were modelled in the monoclinic *P*21/c (CH_2_Cl_2_) or triclinic *P*–1 (THF, DMSO) space group. The THF solvate was best modelled in a unit cell of double the volume of the DMSO species; this is related to the ordering of the lattice solvent molecules and does not substantially affect the chemical environment of the two unique **TBNap** residues. While the DMSO solvate exhibits equivalent packing behavior to the THF species, the lattice DMSO molecules were sufficiently disordered to not be appropriately modelled by even four overlapping orientations and were accounted for using the SQUEEZE routine within PLATON.^[^ ^34^, ^35^ ^]^ The solvent molecules in the CH_2_Cl_2_ solvate also could not be resolved from the diffraction data and were treated similarly. For this discussion, the CH_2_Cl_2_ and DMSO adducts will be directly compared as indicative of each structure type, as both structures have been modelled as solvent‐free, to provide the most appropriate comparison.

**Figure 1:**
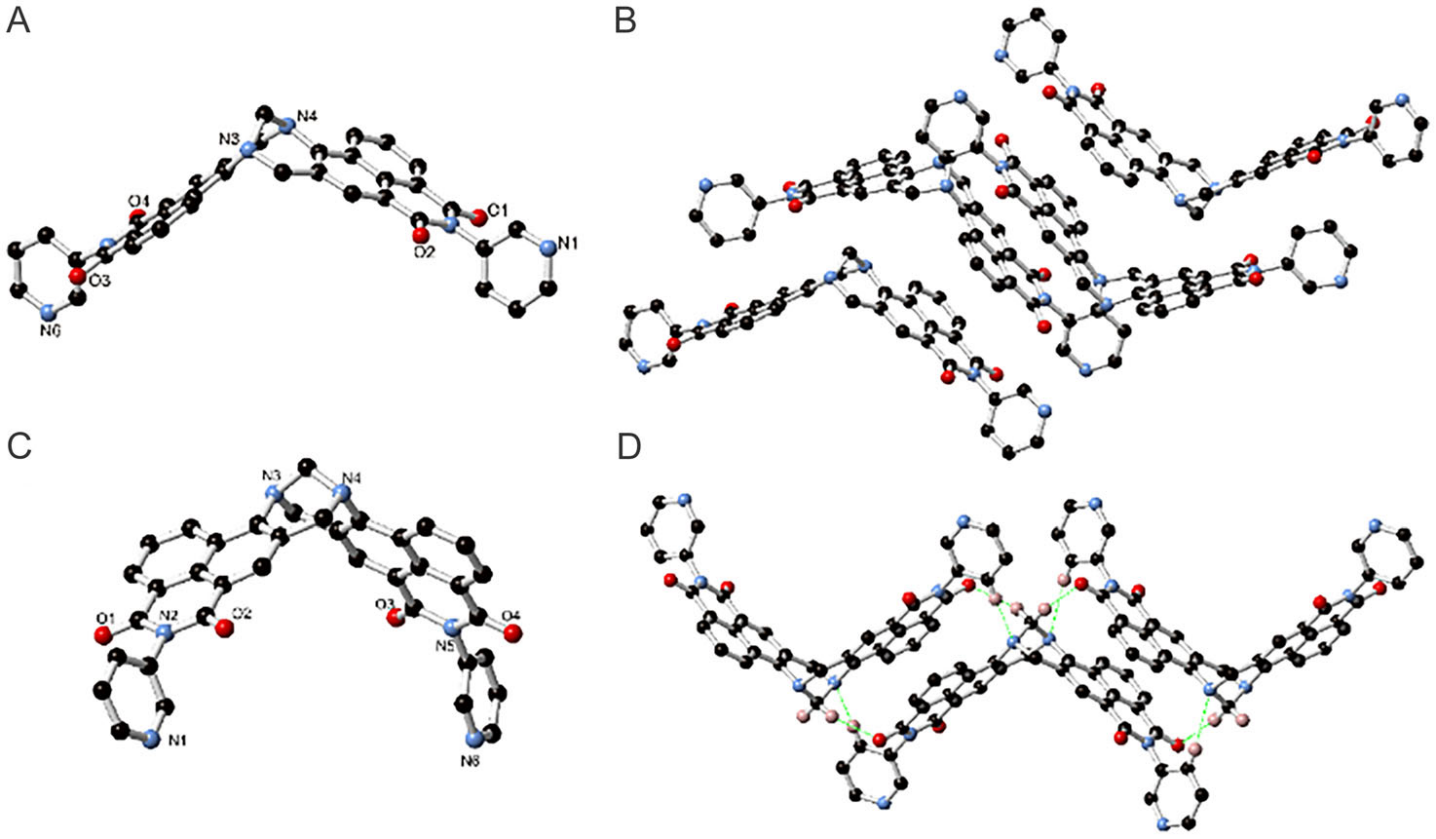
The single‐crystal X‐ray structure of A) a monoclinic solvate of **TBNap** (in CH_2_Cl_2_) and B) stacking interactions between the adjacent molecules. C) The molecular structure of triclinic solvates of **TBNap** (in THF or DMSO) and D) their molecular stacking, highlighting the notable C—H···N contacts from the pyridyl 4‐position and the tertiary amines of the TB core reciprocated by the bridgehead CH_2_.

All three solvates display the expected cleft structure for the **TB** moiety, with interplanar angles (measured between the two naphthyl mean planes) of 115.4, 98.3, and 98.4/97.0 (two unique residues) for the CH_2_Cl_2_, DMSO, and THF solvates, respectively (**Figure** **1**A–D), demonstrating the close to orthogonal (cleft angle) geometries. The three structures also exhibit slight conformational differences in the orientations of the terminal 3‐pyridyl groups; the CH_2_Cl_2_ solvate displays a syn‐anti orientation with respect to the central aperture (Figure 1 A), while the THF species was best modelled with only *syn‐syn* behavior (other orientations, if present, were present at low occupancies), and the DMSO solvate was best modelled as a disordered mixture of *syn‐syn* and *syn‐anti* at an ≈55:45 ratio (Figure 1B). The extended structures of the three solvates can be considered in terms of differences between the monoclinic and triclinic forms, as equivalent behavior is encountered in the triclinic THF and DMSO solvates. In all three materials, the extended structures are dominated by *face‐to‐face* π–π interactions between the naphthalimide groups (Figure 1B,D). In the monoclinic form, the most substantive interaction is a *head‐to‐tail* type interaction between two symmetry‐related convex faces of two equivalent molecules of **TBNap**, with parallel naphthyl mean planes separated by 3.55 Å (Figure 1B). This interaction is only present for one of the two naphthalimide rings within the structure (C21–C32); on the opposite side of the molecule, the convex face undergoes an interaction with a concave face of an adjacent molecule in a twisted head‐to‐head fashion (between the C6–C17 and C21–C32 fragments) (Figure 1B). The two naphthyl groups are offset at an angle of 11.0° in this interaction, and the minimum interatomic distance for this interaction is 3.440(5) Å for C15–C27. No other substantial *face‐to‐face* π–π interactions are observed in the monoclinic form, where the remaining interactions are C–H···O and C–H···π contacts (Figure S10, Supporting Information). One significant such interaction is a C–H···O interaction from C26–O1 with C···O distance 3.147(5) Å. In comparison, the triclinic form exhibits *face‐to‐face* π–π stacking exclusively between convex faces of the molecule, both sides of which engage symmetrically in two parallel *head‐to‐tail* modes with naphthyl–naphthyl interplanar distances of 3.41 and 3.46 Å (indicative for the DMSO solvate) (Figure 1D). The concave faces of each molecule provide a pocket for lattice solvent molecules, disordered in the DMSO case but ordered in the THF case. When considering the C–H···O interactions in the triclinic form, one structural feature of particular interest is evident, which is not observed in the monoclinic form, namely, the interactions involving the bridgehead CH_2_ and attached tertiary amines (Figure 1D). In the triclinic form, and in concert with the symmetric π–π interactions described above, the bridgehead CH_2_ group donates two C–H···O hydrogen bonds to the nearby imide oxygen atoms (C···O distances 3.193(3) and 3.457(2) Å), while the attached 3‐pyridyl groups reciprocate with C–H···N interactions from the pyridyl 4‐(or 2‐, in the disordered case) positions to the tertiary amine nitrogen atoms of the bridgehead group (C···N distances 3.370(3) and 3.586(2) Å) (Figure S9–S10, Supporting Information). This interaction stands out as a packing synthon absent from the monoclinic form.

**Figure 2:**
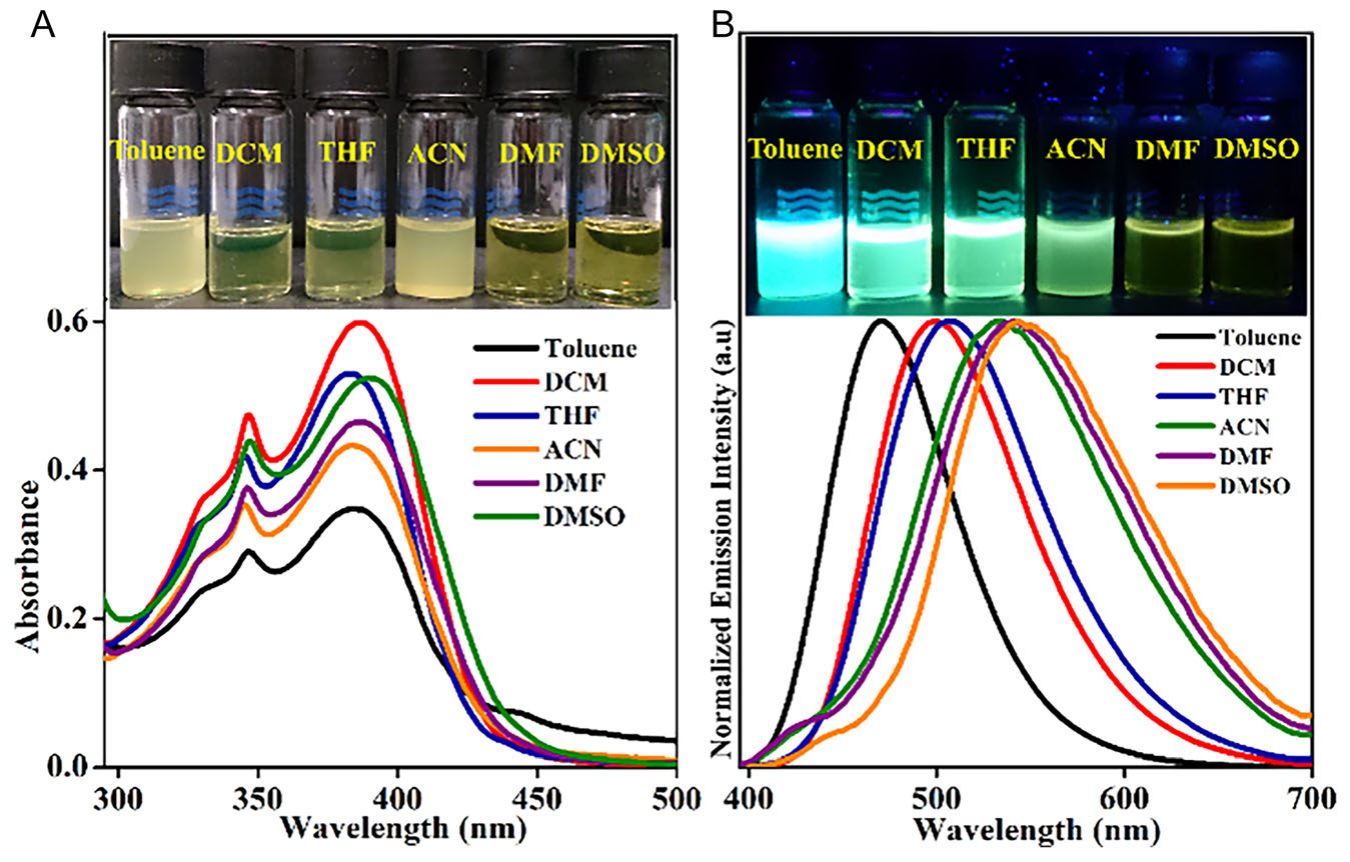
A) The UV–visible absorption spectra and B) normalized fluorescence emission spectra of **TBNap** (1.0 × 10^−6^ M) in solvents with varying polarity (Inset: corresponding photographs).

Amino‐1,8‐naphthalimide derivatives are known to be strongly colored and highly fluorescent due to the ICT transition as mentioned above, which gives rise to a large excited‐state dipole that is dependent on the solvent polarity.^[^ ^36^, ^37^ ^]^ To understand the photophysics, the absorption and emission properties of **TBNap** were analyzed in different solvents with varying polarity (The corresponding photophysical data are summarized in Table S2, Supporting Information). As alluded to above, the **TBNap** was soluble in CH_2_Cl_2_, THF, DMF, and DMSO, but sparingly soluble in toluene and CH_3_CN, and insoluble in H_2_O, even at low (spectroscopic) concentrations. As shown in **Figure** **2**A, the absorption spectra of **TBNap** recorded both in protic and aprotic solvents showed the characteristic low‐energy band at *λ* = 384–391 nm, which is due to the ICT transition, which could be confirmed by solvent‐dependent emission studies. The fluorescence emission spectra of **TBNap** exhibited a remarkable solvatochromism with a significant red shift in the emission maxima with increasing solvent polarity (Figure 2B). For example, **TBNap** displayed blue emission at *λ* = 470 nm in nonpolar toluene, while yellowish‐green emission at *λ* = 544 nm was observed in polar solvent DMSO. We also found that the Stokes shift increased significantly from 4697 to 7193 cm^−1^ (See Table S2, Supporting Information). This was accompanied by an obvious spectral broadening of the emission maximum, upon changing the solvent from nonpolar toluene (solvent polarity index, *P* = 2.4) to polar DMSO (*P* = 7.2). The observed significant red shift in the fluorescence emission maximum and the large Stokes shift with increasing solvent polarity demonstrate that solvent stabilization is more notable in the excited state and confirm that the emission of **TBNap** results from an ICT transition.

**Figure 3:**
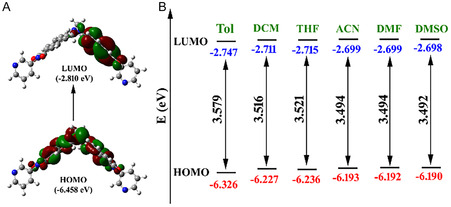
A) The frontier molecular orbitals (HOMO and LUMO) of **TBNap**. B) HOMO and LUMO energy of **TBNap** optimized in different solvents with varying polarity.

To support the existence of ICT‐based emissions, we further performed computational calculations on the **TBNap** structure by employing the density functional theory (DFT) to obtain the optimized structure and determine the frontier molecular orbital energy diagrams. All the DFT calculations were carried out at the B3LYP/6‐311 G(d,p) level using the Gaussian 16 program.^[^ ^38^ ^]^ As it is shown in **Figure** **3**A, the highest occupied molecular orbital (HOMO) of **TBNap** in the gas phase is primarily localized on the **TB** base unit (electron‐rich site acts as a donor), while the lowest unoccupied molecular orbital (LUMO) is largely distributed on the imide moiety (electron‐deficient acts as an acceptor). This confirms that **TBNap** is a typical “*push‐pull”* type fluorophore with ICT‐based transition from **TB** unit to imide moiety upon light irradiation. The structural feature of the energy‐minimized structure of **TBNap** was in good agreement with the structure obtained from the above X‐ray diffraction analysis. Moreover, the experimentally observed solvent‐polarity‐dependent emission changes were validated by further theoretical calculations. **TBNap** was modelled in different solvent systems, and frontier molecular orbitals energy values were determined. As shown in Figure 3B, the HOMO–LUMO energy gap for **TBNap** decreases as the solvent polarity increases. For instance, the HOMO–LUMO energy difference calculated for **TBNap** in nonpolar toluene was 3.579 eV, which was reduced to 3.492 eV for **TBNap** modelled in polar solvent DMSO. This decrease in the energy gap concurs well with the significant red shift observed experimentally when moving from the toluene to the DMSO solvent medium.

We also carried out time‐dependent DFT calculations using CAM‐B3LYP/6‐31 G(d) method to get insights about the absorption and emission phenomena. We included solvent effects using the polarizable continuum model (PCM) approach with THF as a representative medium. Full computational details, including the excited‐state optimization protocols, are provided in the Supporting Information. Our results reveal that the intense and the lowest‐energy absorption band at *λ* = 340 nm corresponds to the S_0_ → S_1_ transition. Orbital analysis shows that this transition is predominantly characterized by HOMO → LUMO excitation. The emission spectrum obtained at the optimized excited state geometry shows a strong fluorescence band at *λ* = 402 nm. The red shift relative to the absorption maximum indicates significant reorganization of the electronic structure and solvent stabilization in the excited state of the **TBNap**.

### 2.2 Solvent‐Guided Morphogenesis of **TBNap**

**Figure 4:**
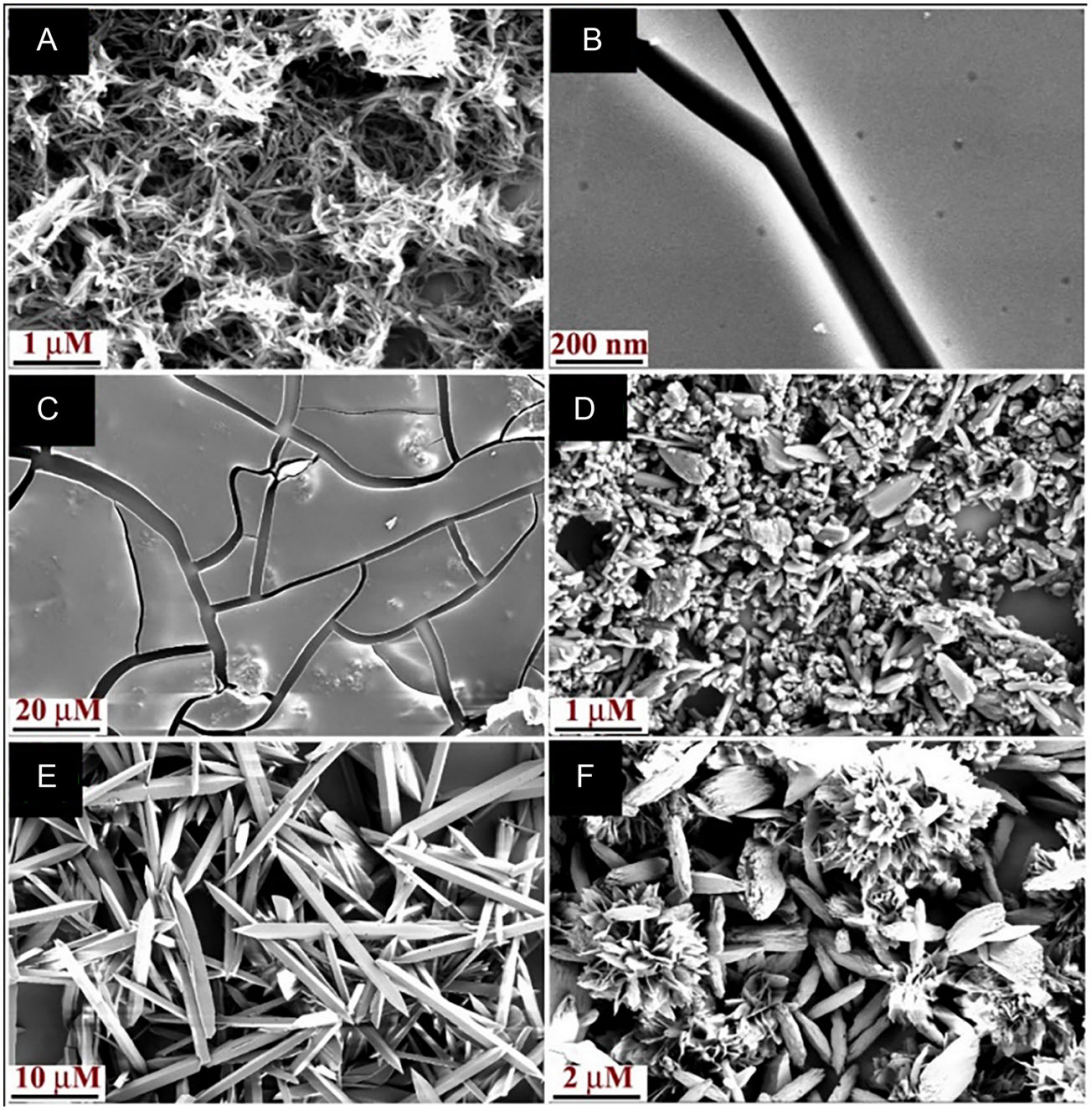
The SEM images of **TBNap** are pictured by dissolving in different solvents with varying polarity. A) Toluene, B) DCM, C) THF, D) ACN, E) DMF, and F) DMSO.

The existence of solvent‐controlled molecular packing and solvent polarity‐dependent positive solvatochromism in different solvents stimulated us to investigate the morphological features of **TBNap** by scanning electron microscopy (SEM) imaging. The SEM samples were prepared by drop casting the solution of **TBNap** (1.0 × 10^−6^ M) in various solvents onto the freshly cleaned silicon surface, followed by natural evaporation at ambient conditions. Six solvents with varying polarity, from nonpolar toluene to polar DMSO, were chosen to probe the effect of solvent polarity on the self‐assembly of **TBNap**. As shown in **Figure** **4**, **TBNap** displayed strikingly different self‐assembled micro or nanostructures with distinctive morphological features under different solvent media. For instance, the CH_2_Cl_2_ and THF solution of **TBNap** showed thin film features with a smooth surface (Figure 4B,C). This can be attributed to its high solubility and fast solvent evaporation, which prevents the essential nucleation for particle formation. While in CH_3_CN, some irregularly shaped submicron particles were formed, possibly caused by the low solubility of **TBNap** that resulted in rapid precipitation of the randomly aggregated particles, presumably due to the concurrent occurrence of nucleation and particle growth (Figure 4D). Gratifyingly, these measurements were all fully reproducible, demonstrating that the morphological features are directly influenced by the nature of the solvent environment and not due to random self‐assembly formation. The results also demonstrate how important “*input*” the nature of the solvent on such assembly formation from a common ligand design, as we outline below.

In the nonpolar solvent toluene, a bundle of a fibrous structure composed of short needles was observed with polydispersity that can be attributed to π–π stacking interaction between toluene and 1,8‐naphthalimide moiety (Figure 4A). However, **TBNap** was self‐assembled into needle‐shaped particles with sharp edges of 10 μM in length when the imaging was done from DMF solution (Figure 4E). While in the DMSO solution, the seed and flower‐like structures with a size in micrometer scale were observed under the same conditions. This indicates that, in addition to the solubility and π–π stacking interactions, the hydrogen bonding ability of polar solvents also plays a crucial role in controlling the morphological features of self‐assembled structures. These findings confirm that the self‐assembly of **TBNap** was intemperately dependent on the solvent medium. Such solvent‐guided self‐assembly of **TBNap** arises from the difference in the extent of solvation of functional moieties present in it. The hydrophobic naphthyl unit is expected to show higher interaction with nonaffinity for polar solvents such as DMF and DMSO. As a result, the solvation of **TBNap** in organic solvents with varying polarity would be significantly different, thus resulting in diverse polar aromatic solvents like toluene, while 3‐pyridyl, **TB**, and imide moieties exhibit stronger self‐assembled structures with distinctive morphological features. This assumption complies well with the X‐ray diffraction analysis of **TBNa**p solvates described above. The extended structure of the DMSO solvate of **TBNap** exhibits, in addition to the symmetrical π–π interactions, multiple hydrogen bonding interactions between the bridgehead CH_2_ group with imide oxygen atoms (C‐H···O) and pyridyl nitrogen atoms (C‐H···N); this particular interaction is absent in the nonpolar CH_2_Cl_2_ solvate of **TBNap**. This study provides a facile route to fine‐tune the morphological features of self‐assembled structures by simply adapting different solvent systems.

### 2.3 AIEE Property of **TBNap**

**Figure 5:**
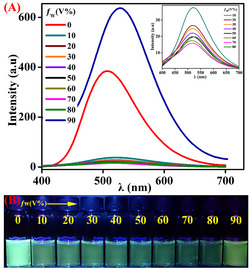
A) The fluorescence emission spectra of **TBNap** in THF/water mixtures with different water fractions (Inset shows the changes in the emission intensity at lower water fractions). B) The corresponding photographs were taken under UV light illumination.

AIE or AIEE is a topical area of research.^[^ ^39^ ^]^ Among various chromophoric ligands, **Nap** derivatives have attracted increased research attention as AIE luminogens for their versatile optoelectronic applications.^[^ ^40^, ^41^ ^]^ As we outlined above, the **Nap** are planar structures that form strong intermolecular π–π stacking interactions in the condensed state can lead to fluorescence quenching (namely, ACQ effect), which limits their potential uses.^[^ ^40^ ^]^ However, we have recently demonstrated that extending the **Nap** at the 4^th^ position with groups such as phenyl carbazole and diphenylamino phenyl groups can lead to AIE‐based systems which show “*on‐off‐on*” behavior, both in solution and within polymers, enabling the delivery of such systems into cells, for use in cellular imaging.^[^ ^42^, ^43^, ^44^, ^45^ ^]^ Hence, the ACQ shortcoming of **Nap** can be addressed by integrating a suitable functional moiety that effectively prevents the strong π–π stacking interactions between the adjacent units and retains the strong fluorescence emission even in an aggregate or condensed state. Given the unique orthogonal nature of the **TBNap** and its highly solvent‐dependent features discussed above, wherein “pure water” emission was quenched, we also analyzed the fluorescence emission spectra of **TBNap** in a THF‐H_2_O binary mixture at room temperature. As shown in **Figure** **5**A, the **TBNap** exhibited a moderate fluorescence emission at *λ* = 507 nm in THF, and as expected, the gradual addition of increased volume percentage (*f*_w_) of poor solvent H_2_O from 10 à 70% induced significant fluorescence emission quenching due to the twisted intramolecular charge transfer (TICT) effect (see Figure 5A inset). However, further increasing the H_2_O fraction from 80 to 90% dramatically enhanced (≈1.7 fold) the fluorescence emission intensity of **TBNap**, which is a typical AIEE property. The most intense fluorescence emission was observed for *f*_w_ = 90% H_2_O with emission maxima at *λ* = 529 nm, accompanied by a 22 nm bathochromic shift. The enhanced fluorescence emission intensity at higher H_2_O content (>80%) was assigned to the restriction of twisted intramolecular rotation due to the formation of the aggregates. TICT prevails, which significantly quenches the fluorescence emission; however, at higher H_2_O content (>80%), AIEE dominates due to the aggregate formation leading to the enhanced fluorescence emission. The notable bathochromic shift in emission maxima of **TBNap** in THF/H_2_O (1:9 by volume) was attributed to the increase in polarity of the medium that stabilizes the excited state dipole, which gives rise to the low‐energy ICT transition, and also due to the effect of a change in self‐assembly. The AIEE feature of **TBNap** was also reflected by noticeable visual color changes from bluish green to colorless to yellowish green upon increased addition of H_2_O (*f*_w_; 10–90%), indicating the naked eye visualization of the AIEE effect (see Figure 5B).

**Figure 6:**
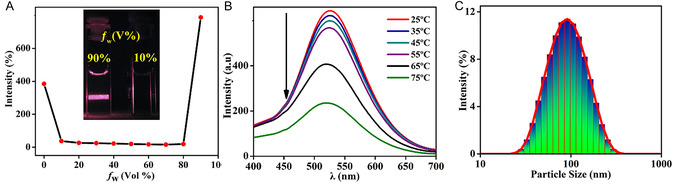
A) The fluorescence intensity of **TBNap** at λ = 507 nm as a function of *f*_w_ (Inset: photos showing the Tyndall effect of **TBNap** in THF with 90% and 10% water fractions. B) Temperature (25 → 75 °C)‐dependent emission characteristics of nanoaggregates of **TBNap** in THF/H_2_O (1:9 v/v) (*λ*_ex_ = 380 nm). C) DLS for **TBNap** measured in THF/H_2_O (10:90% by volume).

To better understand the AIEE feature, a plot of the percentage of emission enhancement versus H_2_O fraction in THF gave a clear trend that the moderate fluorescence emission intensity of **TBNap** in pure THF decreased gradually upon increasing H_2_O content (<80%) and on a further increase of H_2_O fraction (>80%) elicited substantial fluorescence enhancement with the highest being at *f*_w_ = 90% (**Figure** **6**A). The aggregate formation after 90% H_2_O fraction in THF was supported by the Tyndall effect. As shown in Figure 6A inset, no scattering of laser light was observed for a 10% H_2_O fraction in THF, but a 90% H_2_O fraction exhibited scattering of light by aggregate particles in the binary solvent mixture. The use of dynamic light scattering (DLS) revealed that the average diameter of the aggregate in a binary solvent mixture is ≈100 nm (Figure 6C). This confirms the formation of nano‐meter‐sized aggregates at 90% H_2_O fraction in THF. The nanoaggregates formation was further endorsed by observing the self‐assembly formation as a function of temperature. At 25 °C, **TBNap** displayed a strong fluorescence emission in the THF/H_2_O (10:90 by volume fraction) mixture due to the nanoaggregate's formation. Upon gradually increasing the temperature up to 75 °C, the emission intensity of **TBNap** was decreased to more than half of the initial fluorescence emission intensity (Figure 6B). This attenuation of fluorescence emission intensity at higher temperatures is presumably due to the disaggregation of aggregates. This is further evident in the nanoaggregate's formation at 90% H_2_O fraction in THF.

### 2.4 Morphological Study of AIEE Feature of **TBNap**

**Figure 7:**
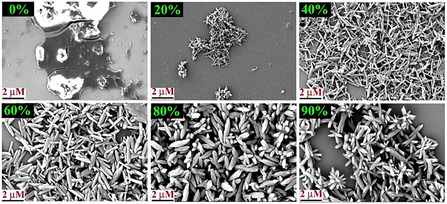
The SEM images of **TBNap** were obtained in THF/H_2_O mixtures with different H_2_O fractions (from 20 to 90%).

**Figure 8:**
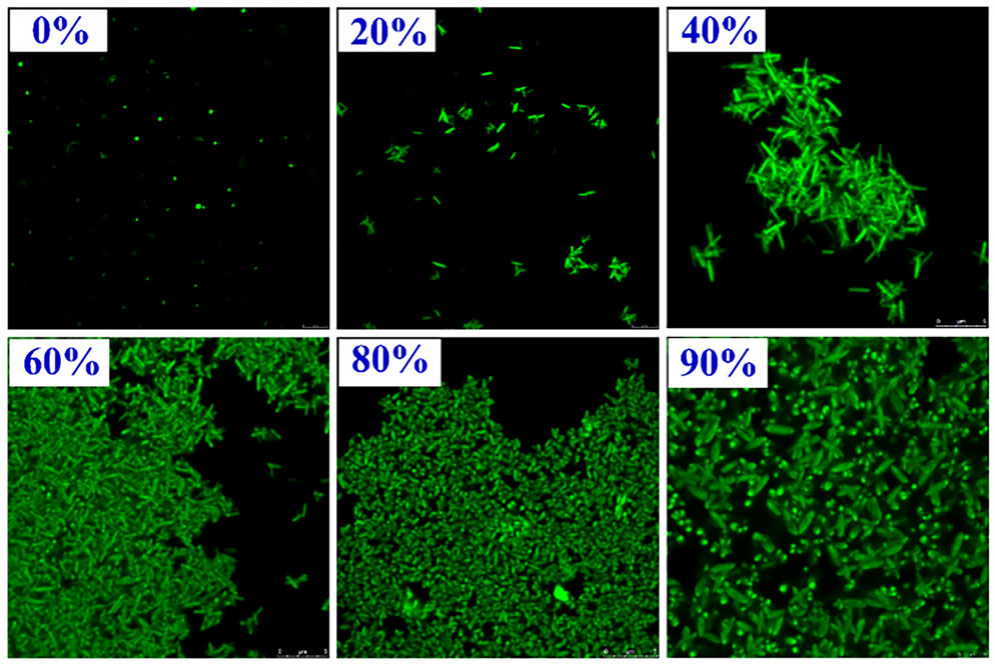
The CLSM images (scale bar: 5 μM) of **TBNap** were obtained in THF/H_2_O mixtures with different H_2_O fractions (from 20 to 90%). *λ*_exc_ = 405 nm.

The positive solvatochromism, solvent‐guided morphogenesis, and AIEE property in a binary solvent mixture of **TBNap** evident that solvent polarity and solute‐solvent interactions play the predominant role in controlling the molecular aggregation of **TBNap**. Therefore, solvent‐dependent aggregation can be exploited to tune and visualize the morphological features of AIEE aggregates. To comprehend the morphology of aggregates formed during the AIEE process, the SEM images of samples obtained after different volume percentages of H_2_O were recorded. The samples for the SEM imaging study were prepared by drop‐casting 5 μL solution of **TBNap** (1.0 × 10^−6^ M) in THF with varied H_2_O fractions onto the silicon surface, followed by slow solvent evaporation at ambient conditions. As shown in **Figure** **7**, the SEM imaging analysis confirmed the nanoaggregate formation through solvent‐polarity‐dependent evolutionary morphogenesis. The THF solution of **TBNap** appeared as a thin film with a smooth surface. Upon 20% H_2_O mixing to the THF solution, the thin film switched to needle‐shaped aggregates, which continued growing into nanorods at 40–60% H_2_O fraction. Then transformed to a typical rice‐like morphology with soft edges at 80% H_2_O fraction. Interestingly, at 90% H_2_O fraction in THF, **TBNap** exhibited a “flower‐like” morphology in which multiple nanorods were inextricably intertwined. Notably, these SEM images of different morphological features of **TBNap** were stable and fully reproducible. The obtained SEM images of the AIEE solution of **TBNap** demonstrate that solvent polarity can be exploited to tune the morphology of aggregates and thus their morphology‐dependent functional properties. Given the strong fluorescence property of **TBNap**, confocal laser scanning microscopy (CLSM) imaging was further employed to visualize the morphological features of AIEE aggregates. As depicted in **Figure** **8**, fluorescent nanoaggregates were formed, and at 90% H_2_O fraction, highly emissive green‐fluorescent aggregates with flower‐like morphology appeared. But, in a binary THF/H_2_O solvent mixture with different volume percentages of H_2_O, then in pure THF solution, the **TBNap** forms a nonemissive thin film with almost no particles

### 2.5 In Situ, Real‐Time Visual Imaging of the AIEE Feature of **TBNap**

**Figure 9:**
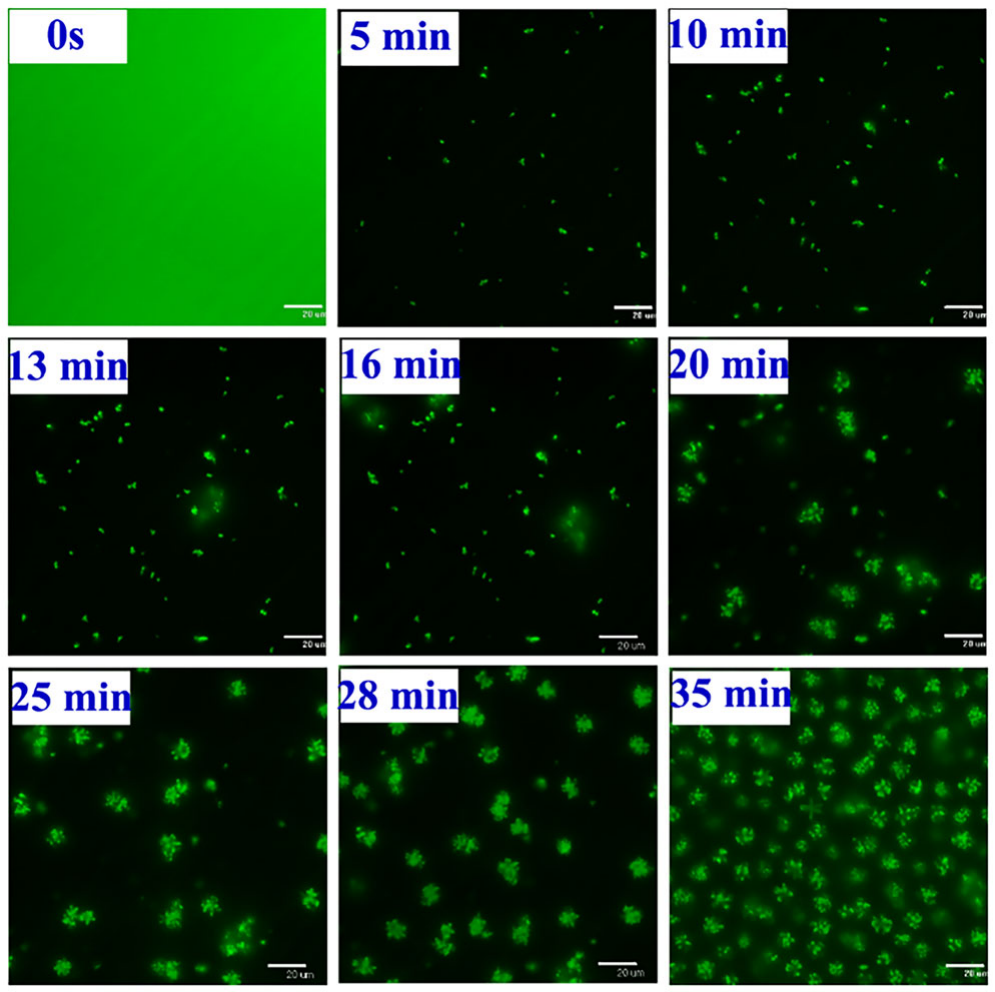
Time (0s → 35 min)‐dependent CLSM images (scale bar: 20 μM) of **TBNap** in THF/H_2_O (9:1 by volume ratio) show the progress of AIEE aggregate formations. All the snapshots are taken from the supplementary movie.

The solvent polarity‐dependent morphogenesis and distinct morphological outcome of AIEE aggregates of **TBNap** motivated us to investigate further the in situ, real‐time visualization of the AIEE aggregate formation in solution using confocal fluorescence microscopy. To this end, the THF solution of **TBNap** (1 × 10^−6^ M) was taken in a specially designed bent‐shaped quartz tube, and to this THF solution, 90% of H_2_O by volume ratio was added, and then aggregates formation was monitored in live over time (0 to 35 min) at 22 °C upon exciting at *λ*_ex_ = 405 nm, and observing the self‐assembly formation in situ. Snapshots taken at different time intervals are summarized in **Figure** **9** and demonstrate the observed self‐assembly formation in real time, where the AIEE is employed to “watch” the process. As can be seen in Figure 9A, before adding H_2_O, at *t* = 0s, the clear THF solution of **TBNap** showed green fluorescence emission emanating from the ICT transition. No aggregates or particles are formed at *t* = 0s in pure THF solution. After the addition of 90% H_2_O, the green fluorescence emission of **TBNap** in THF was quenched completely, along with the appearance of anisotropic tiny particles. Until *t* = 10 min after H_2_O addition, confocal imaging showed no evidence for the formation of fluorescent aggregates of **TBNap**. At *t* = 13 min, the confocal images revealed the appearance of star‐like emissive meta‐stable nanoaggregates in a binary solvent medium. This indicates that the **TBNap** monomer required a minimum of 10 min for the nucleation processes to form AIEE nanoaggregates. Afterward, the AIEE aggregates were seen to appear in large numbers and continue to grow or “*develop*” in solution.

As time passed, more fluorescent aggregates kept forming; as is evident at *t* = 35 min, the **TBNap** monomers were transformed into stable fluorescent nanoaggregates of varied sizes, the accordance with the SEM and CLSM images (the obtained supplementary movie is attached in the supporting file, see Supporting Information). This study demonstrates the potential of using the CLSM technique for the in situ real‐time visual imaging of fluorescent nanoaggregate formation in native conditions. It also shows that the morphology of the resulting aggregates is the same as that observed from drop‐cast samples that were imaged using SEM. Clearly demonstrating that the self‐assembly morphological feature of aggregates obtained from in situ visual imaging was in good process is “programmable” by modulating the experimental conditions, is occurring in the solution, and (in the case of the SEM) is not due to evaporation events.

## 3 Conclusion

Here, we developed a unique luminogen **TBNap** structure with orthogonal structural properties and demonstrated its solvent‐guided morphogenesis and real‐time visual imaging of AIEE nanoaggregate formation in a binary solvent medium. This **TBNap** structure was synthesized in high yield and characterized using standard spectroscopic techniques, including X‐ray diffraction analysis. A positive solvatochromism in different solvents with increasing polarity and solvent‐polarity‐dependent nano‐ and microstructures of **TBNap** was imaged using SEM and CLSM microscopies. Furthermore, the AIEE process was monitored in real‐time using CLSM, and at different time intervals, the morphology of nanoaggregates emanating from AIEE was imaged. This study provided a deeper understanding of self‐assembled nanoaggregate formation in native conditions. As such, this is an important breakthrough and advancement in the molecular self‐assembly processes, allowing us to control and manage the self‐assembly process at the molecular level and develop technologically useful new functional materials and responsive structures with desired functions. Further work is in progress on exploring the fluorescence sensing properties of **TBNap** toward emerging organic contaminants and toxic environmental pollutants. Also, **TBNap** will be functionalized with suitable groups to make it water‐soluble for its biological applications.

## 4 Experimental Section

4.1

4.1.1

##### Materials and Methods

All solvents and chemicals were purchased from various commercial sources and used without further purification. Solvents used were high‐performance liquid chromatography (HPLC) grade unless otherwise stated. 4‐Nitro‐1,8‐naphthalic anhydride, 3‐aminopyridine, Pd/C (10 wt% loading), paraformaldehyde, and trifluoroacetic acid (TFA) were purchased from various commercial sources and used as received. Deuterated solvents [(CD_3_)_2_SO and CD_2_Cl_2_] used for NMR analyses were purchased from Sigma‐Aldrich or Apollo Scientific. *N*‐(3‐pyridyl)‐4‐nitro‐1,8‐naphthalimide was synthesized following the procedure reported in the literature.^[^ ^32^ ^]^

The melting point was determined using an electrochemical IA9000 digital melting point apparatus in an unsealed capillary tube. The elemental analysis for C, H, and N was performed on an Exeter analytical CE‐450 elemental analyzer at the Microanalytical Laboratory, University College Dublin (UCD). FTIR spectra (4 000 400 cm^−1^) were recorded on a Perkin‐Elmer spectrometer with a universal attenuated total reflectance (ATR) sampling accessory. All NMR spectra were recorded on a Bruker‐DPX‐400‐Avance spectrometer operating at 400 MHz for ^1^H NMR and 101 MHz for ^13^C NMR in a commercially available deuterated solvent. Chemical shifts are reported in parts‐per‐million (ppm) relative to the internal solvent (CD_3_)_2_SO = 2.5 ppm signal. All NMR data were processed with Bruker Win‐NMR 5.0, Topspin, and MestReNova software. Multiplicities were abbreviated as follows: singlet (s), doublet (d), doublet of doublet (dd), triplet (t), multiplet (m). Electrospray ionization (ESI) mass spectra were acquired on a Bruker microTOF‐Q III spectrometer interfaced to a Dionex UltiMate 3000 LC or direct insertion probe. The instrument was operated in positive or negative mode as required. Agilent tuning mix APCI‐TOF was used to calibrate the system. The *m/z* values were recorded over a range of 100–1600. MicroTof control and HyStar software were used to carry out the analysis. HPLC‐grade CH_3_CN or CH_3_OH was used as the carrier solvent. UV–visible absorption spectra were recorded in 1 cm quartz cuvettes (Hellma) on a Varian Cary 50 spectrometer. Baseline correction was applied for all spectra. Emission spectra were recorded on a Varian Cary Eclipse Fluorimeter. The temperature was kept constant, except for the temperature‐dependent emission studies, throughout the measurements at 298 K using a thermostat unit block. Morphological features were imaged by field‐emission SEM using Zeiss ULTRA Plus with an SE2 or in‐lens detector in the Advanced Microscopy Laboratory, CRANN, Trinity College Dublin. The samples were prepared by drop‐casting **TBNap** on silica wafers, then coated with Au and dried under vacuum before the imaging. Confocal fluorescence microscopy images were obtained using Leica SP8 STED confocal microscopy with a 40X oil immersion lens. Image analysis was performed using FluoView Version 7.1 Software.

##### Synthesis and Characterization: N‐(3‐Pyridyl)‐4‐Amino‐1,8‐Naphthalimide

*N*‐(3‐Pyridyl)‐4‐nitro‐1,8‐naphthalimide (200 mg, 0.63 mmol) was reduced by catalytic hydrogenation using Pd/C (10%, 20 mg) at 3 atm of H_2_ in ethanol (15 mL) for 12 h. The mixture was diluted with DCM‐CH_3_OH (1:1, 400 mL) and stirred in the dark for 1 h. The bright green reaction mixture was filtered through celite and washed several times with DCM‐CH_3_OH (1:1). The solvents were removed under reduced pressure to isolate *N*‐(3‐pyridyl)‐4‐amino‐1,8‐naphthalimide as an orange solid (116 mg, 0.40 mmol, 64%) after trituration with cold‐diethyl ether. Melting point 315–318 °C (decomp.). Anal. Calcd (%) for C_17_H_11_N_3_O_2_·0.35CH_3_OH: C, 69.35; H, 4.16; N, 13.98: Found C, 69.01; H, 3.69; N, 13.99. HRMS (*m/z*): calcd for C_17_H_12_N_3_O_2_ 290.0930; found 290.0938 [M + H]^+^. ^1^H NMR (400 MHz, (CD_3_)_2_SO) *δ* 8.65 (d, *J* = 8.3 Hz, 1H), 8.58 (d, *J* = 4.7 Hz, 1 H), 8.50 (d, *J* = 2.1 Hz, 1H), 8.41 (d, *J* = 7.2 Hz, 1 H), 8.17 (d, *J* = 8.4 Hz, 1 H), 7.79 (d, *J* = 8.1Hz, 1H), 7.66 (t, *J* = 7.8 Hz, 1H), 7.53 (t, *J* = 6.3 Hz, 3 H), 6.85 (d, *J* = 8.4 Hz, 1H); ^13^C NMR (101 MHz, (CD_3_)_2_SO) *δ* 164.47, 163.48, 153.53, 150.40, 149.09, 137.54, 134.60, 133.72, 131.70, 130.76, 130.17, 124.45, 124.21, 122.44, 119.94, 108.68, 107.87. FTIR *υ*_max_ (ATR, cm^−1^) 3418, 3327, 3213, 1687, 1653, 1639, 1618, 1576, 1530, 1482, 1425, 1402, 1367, 1351, 1306, 1265, 1236, 1196, 1173, 1148, 1109, 1029, 956, 913, 847, 835, 824, 798, 775, 754, 732, 706, 677, 659, 623, 594, 578, 510, 559, 543.

##### Synthesis and Characterization: Bis‐[N‐(3‐Pyridyl)]‐9,18‐Methano‐1,8‐Naphthalimido[b,f][1,5]diazocine (**TBNap**)

A mixture of *N*‐(3‐pyridyl)‐4‐amino‐1,8‐naphthalimide (150 mg, 0.52 mmol, 1.0 eq.) and paraformaldehyde (23.3 mg, 0.78 mmol, 1.5 eq.) in neat TFA (4 mL) was stirred at room temperature for 12 h under a nitrogen atmosphere. The reaction mixture was then neutralized and further basified to pH = ≈12 by the slow addition of aqueous ammonia. The aqueous solution was extracted several times with CH_2_Cl_2_ and the organic extract was washed with saturated NaHCO_3_ (2 × 50 mL), brine solution (2 × 50 mL) followed by H_2_O (1 × 100 mL). The combined filtrates were removed under reduced pressure and trituration with cold‐diethyl ether giving the expected product **TBNap** (130 mg, 0.21 mmol, 81%) as a bright yellow solid. Melting point 355–358 °C (decomp.). Anal. Calcd (%) for C_37_H_22_N_6_O_4_·0.6CH_2_Cl_2_: C, 67.85; H, 3.51; N, 12.63: Found C, 67.92; H, 3.40; N, 12.80. HRMS (*m/z*): calcd for C_37_H_23_N_6_O_4_ 615.1781; found 615.1770 [M + H]^+^. ^1^H NMR (400 MHz, (CD_3_)_2_SO) *δ* 8.78 (d, *J* = 8.5 Hz, 2 H), 8.60 (dd, *J* = 4.8, 1.5 Hz, 2 H), 8.50 (s, 2 H), 8.48 (s, 1 H), 8.14 (s, 2 H), 8.02–7.95 (m, 2 H), 7.78 (d, *J* = 7.8 Hz, 2 H), 7.54 (dd, *J* = 8.2, 5.1 Hz, 2 H), 5.20 (d, *J* = 17.4 Hz, 2 H), 4.77 (s, 2 H), 4.69 (d, *J* = 17.5 Hz, 2 H); ^13^C NMR (101 MHz, CD_2_Cl_2_) *δ* 163.90, 163.40, 149.78, 149.61, 149.23, 136.30, 132.16, 131.02, 130.78, 129.36, 128.53, 127.47, 127.03, 125.63, 123.54, 122.82, 118.33, 66.86, 57.03. FTIR *υ*_max_ (ATR, cm^−1^) 3082, 1703, 1664, 1595, 1571, 1510, 1459, 1426, 1402, 1371, 1352, 1299, 1245, 1176, 1128, 1047, 1032, 996, 956, 927, 883, 782, 771, 754, 719, 677, 617, 586, 552.

##### Synthesis and Characterization: Single‐Crystal X‐Ray Diffraction Experimental Details

All single‐crystal X‐ray diffraction data were collected on a Bruker APEX‐II DUO diffractometer using microfocus Cu Kα (λ = 1.5405 Å) or Mo Kα (*λ* = 0.71073 Å) radiation. All data collections were carried out using standard ω and *φ* scans at 100 K with temperature control provided by a Cobra cryostream. Data reduction and multi‐scan absorption corrections were applied using SADABS^[^ ^46^ ^]^ within the Bruker APEX3 software suite.^[^ ^47^ ^]^ The data sets were solved using the intrinsic phasing routine with SHELXT^[^ ^48^ ^]^ and refined on F2 using least squares techniques with SHELXL^[^ ^49^ ^]^ operating within the OLEX‐2 GUI.^[^ ^50^ ^]^ Nonhydrogen atoms were located from their residuals within the Fourier difference map. In contrast, hydrogen atoms were placed in calculated positions with Uiso dependencies derived from their carrier atoms. For the DMSO and DCM adducts of **TBNap**, the lattice solvent molecules could not be satisfactorily localized in the structural model for a chemically sensible description of the overall solvation; in both cases, the solvent molecule was susceptible to localized tumbling within a narrow, roughly spherical pocket within the structure. The SQUEEZE routine^[^ ^51^ ^]^ was deployed in these cases to remove the contribution of these regions from the measured structure factors and allow for a more meaningful refinement of the **TBNap** atom positions.

## Supporting Information

Supporting Information is available from the Wiley Online Library or from the author.

## Conflict of Interest

The authors declare no conflict of interest.

## Supporting information

Supplementary Material

## Data Availability

The data that support the findings of this study are available from the corresponding author upon reasonable request.
