# Supplementary material for: Real‐Time In Situ Imaging of Aggregation‐Induced Emission and Solvent‐Guided Morphogenesis of a “V‐Shaped” 4‐Amino‐1,8‐Naphthalimide Tröger's Base Supramolecular Scaffold
Source: Small Sci. 2025 Oct 21;5(12):e202500386. doi: 10.1002/smsc.202500386 (PMC12697783; doi:10.1002/smsc.202500386)
Supplement: Supplementary file 1 — Supplementary Material [file SMSC-5-e202500386-s001.zip › smsc202500386-sup-0001-SuppData-S1.pdf]

Supporting Information

Real-Time *In-Situ* Imaging of Aggregation-Induced Emission and Solvent-Guided Morphogenesis of A 'V-Shaped' 4-Amino-1,8-Naphthalimide Tröger's base Supramolecular Scaffold

Sankarasekaran Shanmugaraju,*[a,b] Deivasigamani Umadevi,[b] Aramballi J. Savyasachi,[a] Chris S. Hawes,[c] Jonathan A. Kitchen,[d] Gavin J. McManus,[e] and Thorfinnur Gunnlaugsson*[a]

[a]School of Chemistry and Trinity Biomedical Sciences Institute (TBSI), Trinity College Dublin, The University of Dublin, Dublin 2, Ireland. E-mail: gunnlaut@tcd.ie

[b]Department of Chemistry, Indian Institute of Technology Palakkad, Palakkad-678623, Kerala, India. E-mail: shanmugam@iitpkd.ac.in

[c]School of Chemical and Physical Sciences, Keele University, Keele, ST5 5BG UK.

[d]Chemistry, Institute of Natural and Mathematical Sciences, Massey University, Auckland 0632, New Zealand.

[e]School of Biochemistry and Immunology, Trinity Biomedical Sciences Institute (TBSI), Trinity College Dublin, The University of Dublin, Dublin 2, Ireland.

Table of Contents: **Page No**

FT-IR spectrum of **Nap** 2
FT-IR spectrum of **TBNap** 2
^{1}H NMR spectrum of **Nap** 3
^{13}C NMR spectrum of **Nap** 3
^{1}H NMR spectrum of **TBNap** 4
^{13}C NMR spectrum of **TBNap** 4
HRMS spectrum of **Nap** 5
HRMS spectrum of **TBNap** 5
Solid-state packing view of a monoclinic CH_2Cl_2 solvate of **TBNap** 5
Solid-state packing of the view of triclinic THF or DMSO solvates of **TBNap** 6
Crystal and refinement parameters for all structures 7
Photophysical properties of **TBNap** measured in different solvents 7

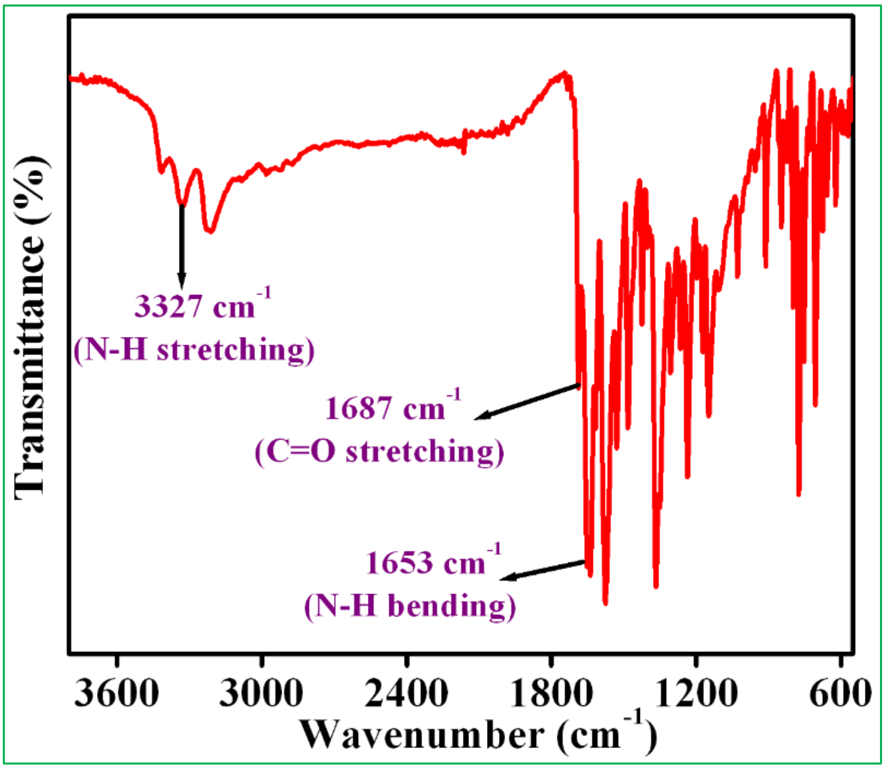

Figure S1 | FT-IR spectrum of **Nap**.

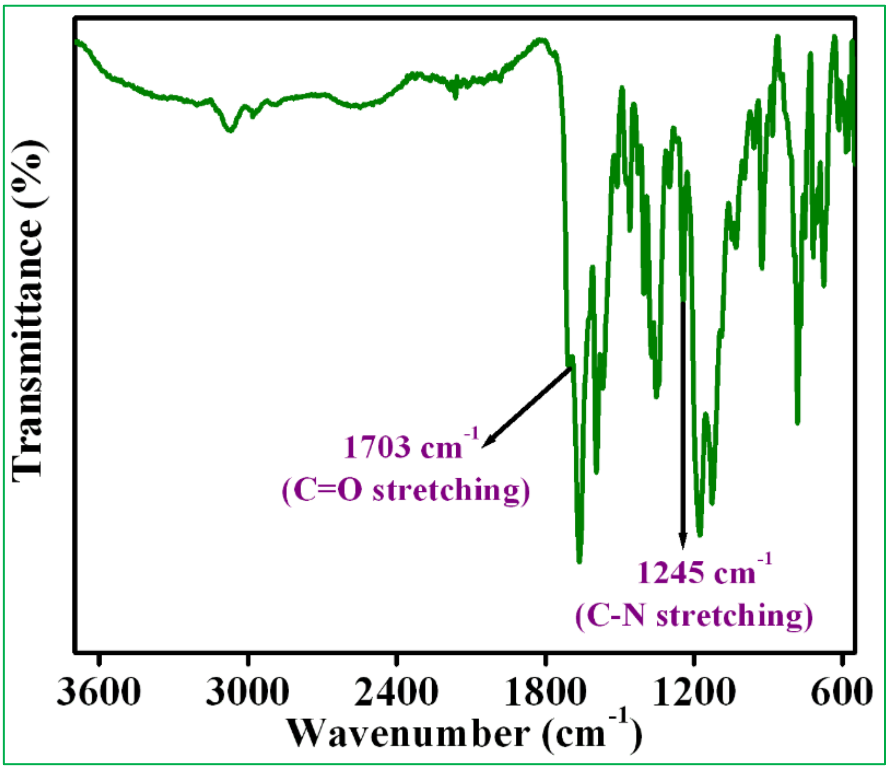

Figure S2 | FT-IR spectrum of **TBNap**.

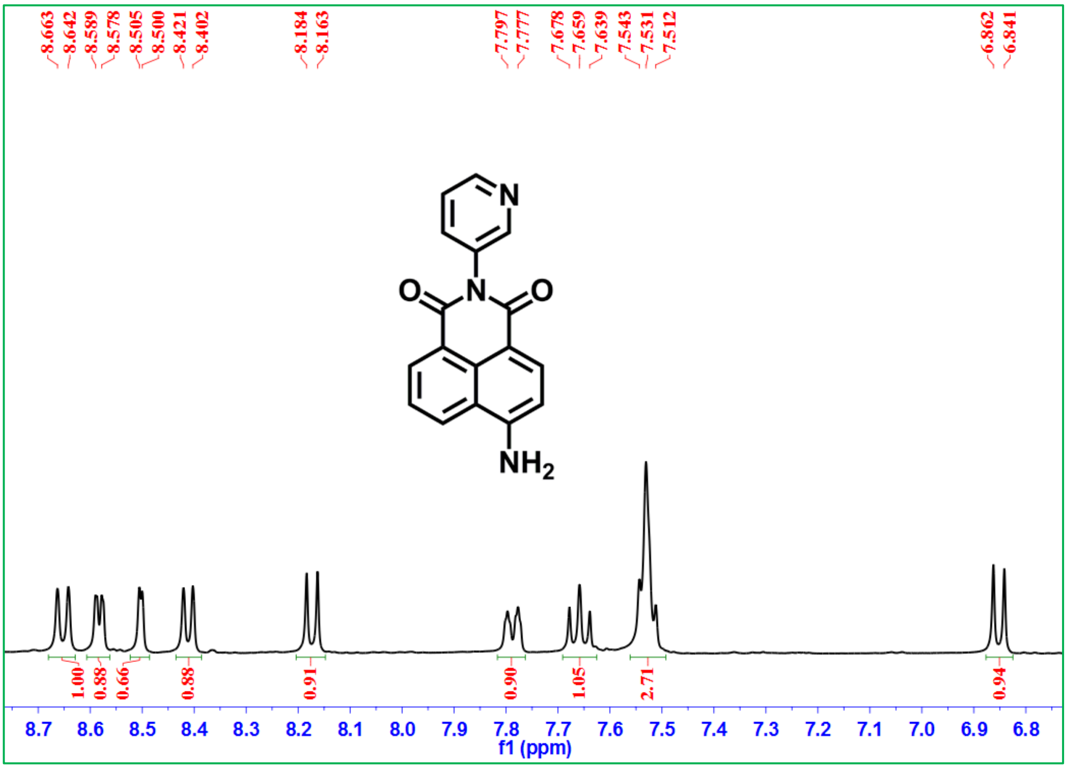

Figure S3 | ^{1}H NMR spectrum of **Nap** (400 MHz, $(CD_3)_2SO$).

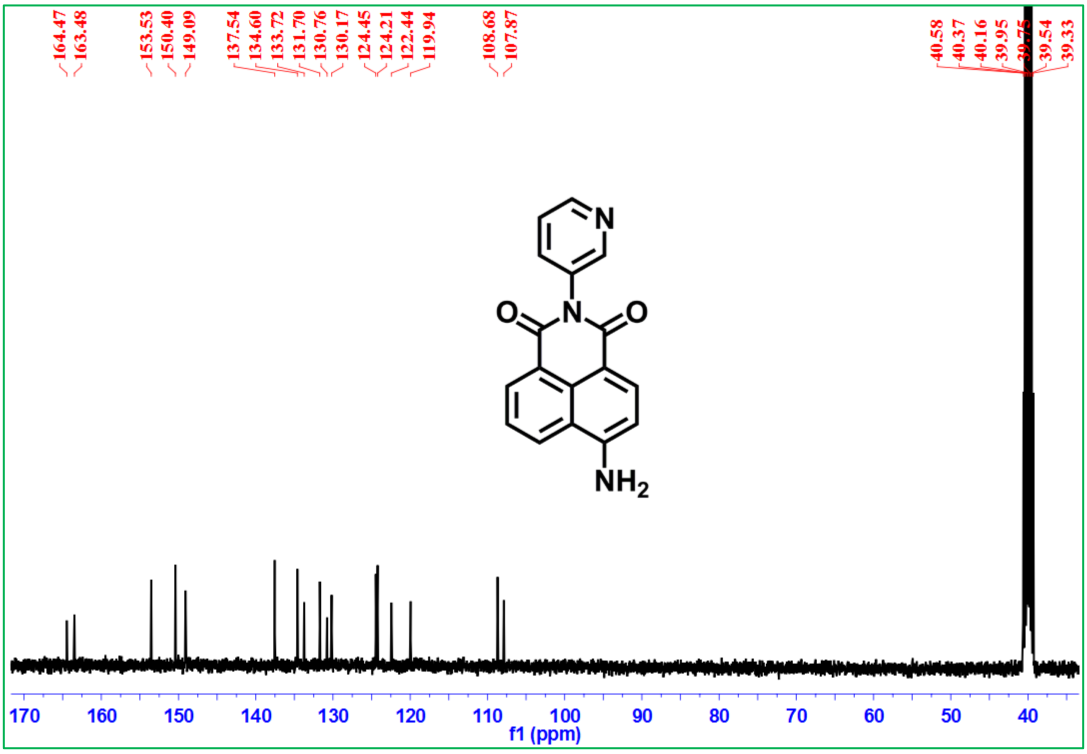

Figure S4 | ^{13}C NMR spectrum of **Nap** (101 MHz, $(CD_3)_2SO$).

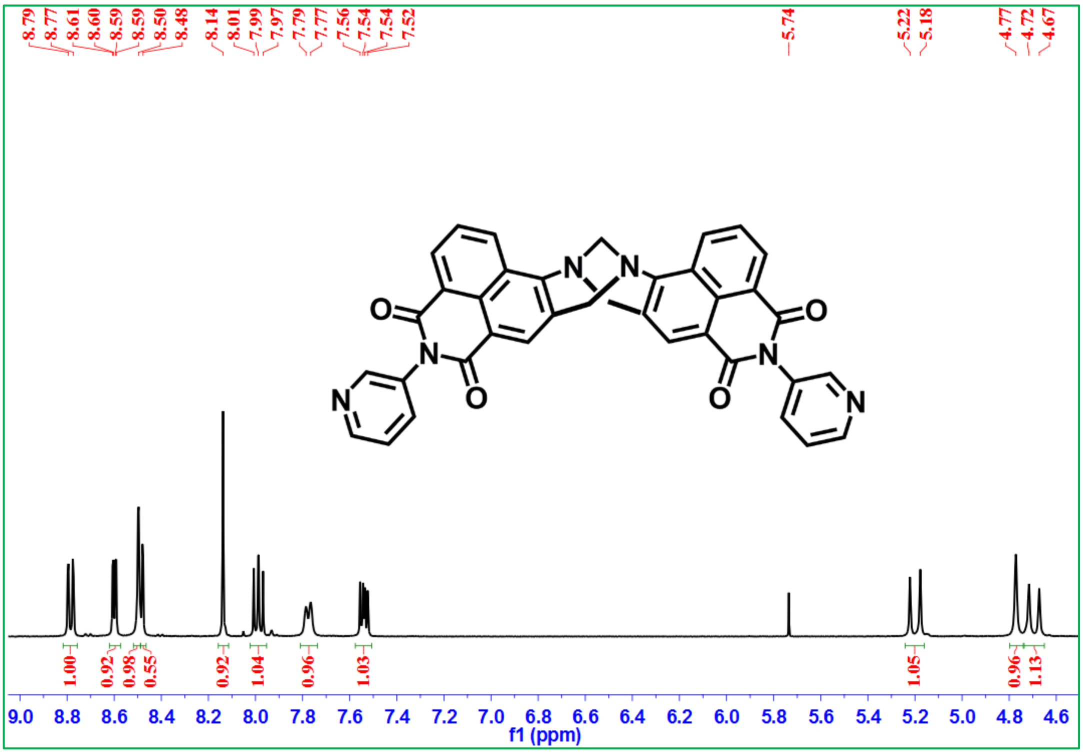

Figure S5 | ^{1}H NMR spectrum of **TBNap** (400 MHz, $(CD_3)_2SO$).

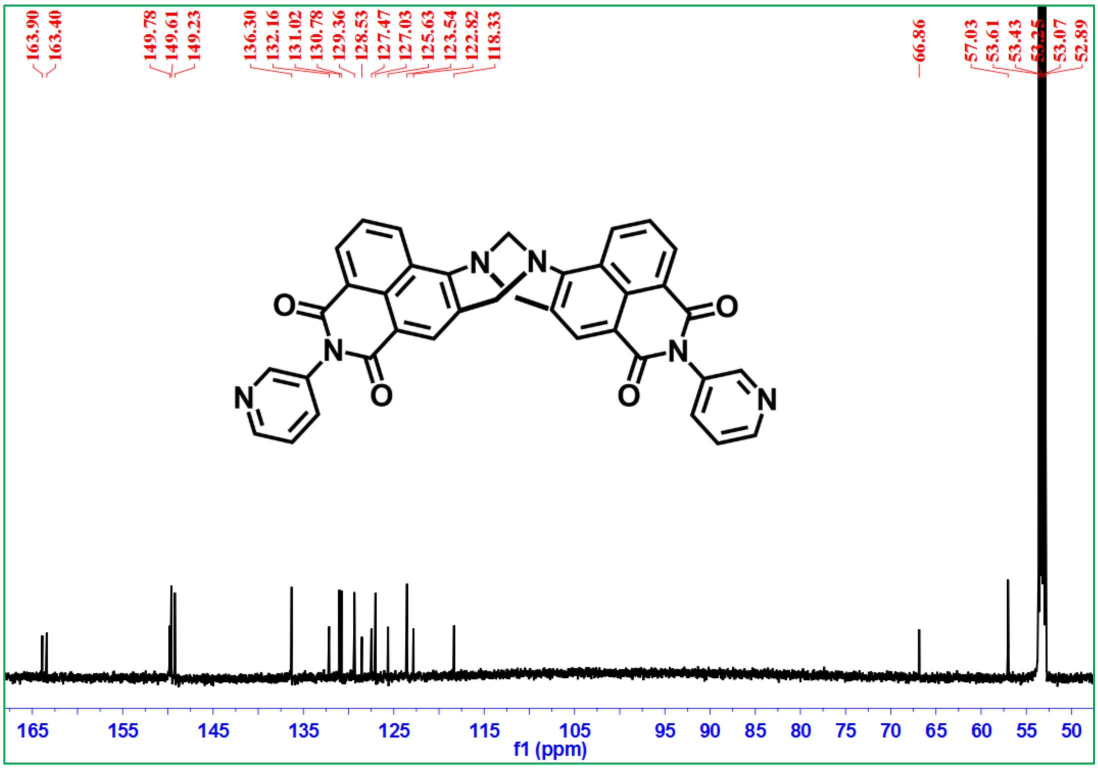

Figure S6 | ^{13}C NMR spectrum of **TBNap** (101 MHz, (CD_2Cl_2)).

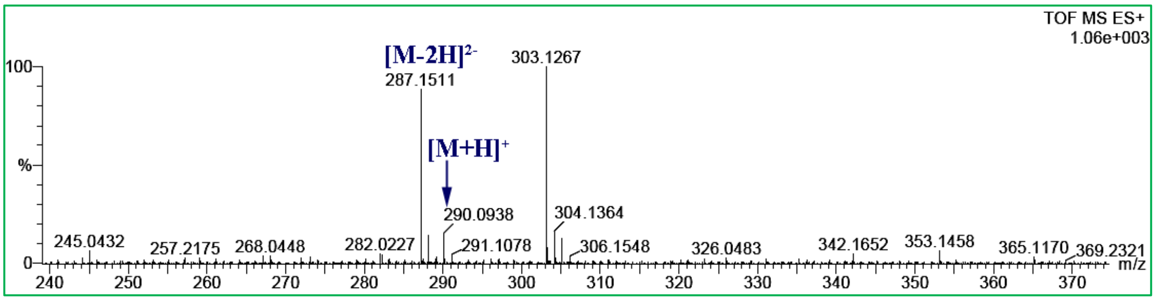

Figure S7 | HRMS spectrum of **Nap**.

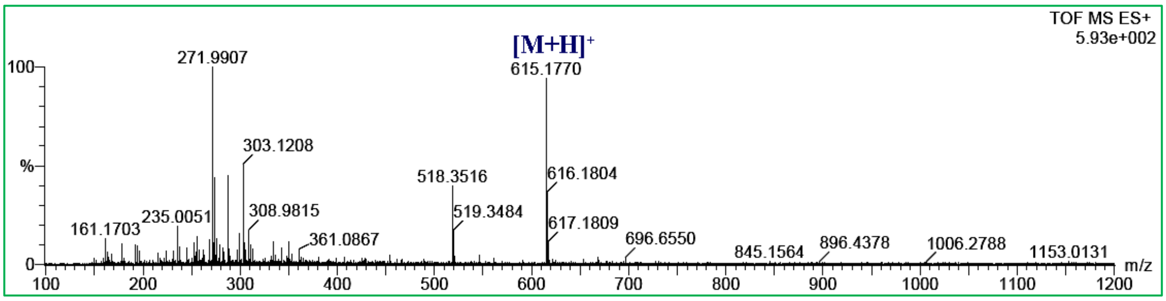

Figure S8 | HRMS spectrum of **TBNap**.

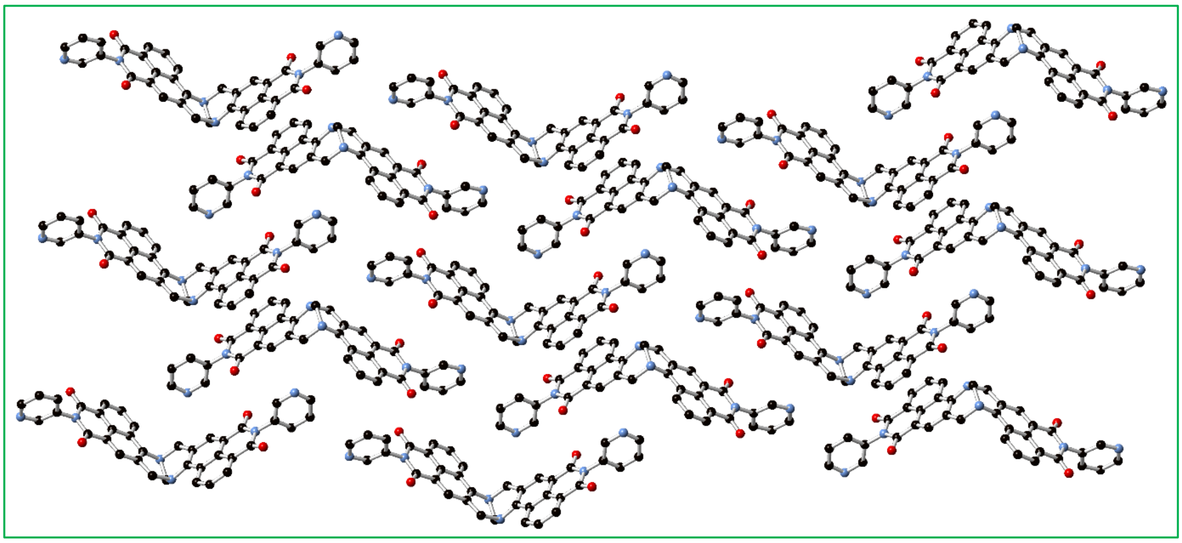

Figure S9 | The extended structure of the CH_2Cl_2 solvate of **TBNap** is viewed parallel to the crystallographic *a*-axis. Hydrogen atoms are omitted for clarity.

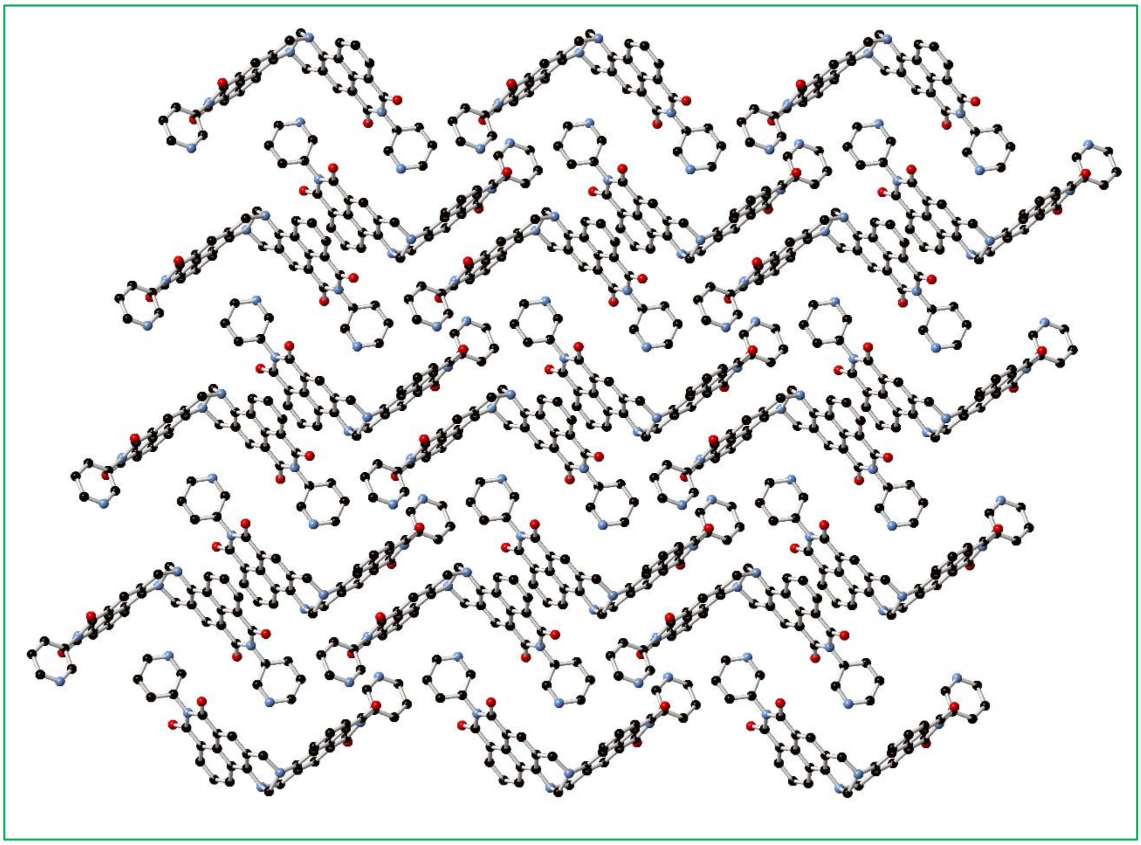

Figure S10 | The extended structure of THF or DMSO solvate of **TBNap** viewed parallel to the crystallographic *c*-axis. Hydrogen atoms and lattice solvent disorder are omitted for clarity.

Table S1. Crystal and refinement parameters for all structures.

Identification code	**L_DCM**	**L_DMSO**	**L_THF**
Empirical formula	$C_{37}H_{22}N_6O_4$	$C_{37}H_{22}N_6O_4$	$C_{41}H_{30}N_6O_5$
Formula weight	614.6	614.58	686.71
Temperature/K	100	100	100
Crystal system	monoclinic	triclinic	triclinic
Space group	$P2_1/c$	*P*-1	*P*-1
a/Å	11.693(7)	10.5924(4)	10.5355(4)
b/Å	21.478(13)	11.2345(4)	16.2440(5)
c/Å	11.912(7)	13.8583(5)	19.5136(6)
α/°	90	79.527(2)	103.068(2)
β/°	90.341(14)	86.382(2)	98.422(2)
γ/°	90	80.665(2)	93.821(2)
Volume/Å^3	2992(3)	1599.23(10)	3200.67(19)
Z	4	2	4
ρ_{calc}g/cm^3	1.365	1.276	1.425
μ/mm^{-1}	0.092	0.7	0.783
F(000)	1272	636	1432

Crystal size/mm^3	0.21 × 0.13 × 0.12	0.15 × 0.09 × 0.06	0.15 × 0.13 × 0.07
Radiation	MoKα (λ = 0.71075)	CuKα (λ = 1.54178)	CuKα (λ = 1.54178)
2Θ range for data collection/°	5.108 to 49.998	6.49 to 136.718	6.412 to 136.622
Index ranges	-13 ≤ h ≤ 13, -25 ≤ k ≤ 25, -14 ≤ l ≤ 9	-11 ≤ h ≤ 12, -13 ≤ k ≤ 13, -16 ≤ l ≤ 16	-12 ≤ h ≤ 12, -19 ≤ k ≤ 19, -23 ≤ l ≤ 22
Reflections collected	28578	14845	25769
Independent reflections	5255 [R_{int} = 0.0396, R_{sigma} = 0.0300]	5787 [R_{int} = 0.0320, R_{sigma} = 0.0377]	11579 [R_{int} = 0.0362, R_{sigma} = 0.0489]
Reflections Observed [I>=2σ (I)]	4613	4646	8047
Data/restraints/parameters	5255/0/424	5787/0/426	11579/9/946
Goodness-of-fit on F^2	1.203	1.062	1.035
Final R indexes [I>=2σ (I)]	R_1 = 0.0904, wR_2 = 0.2290	R_1 = 0.0479, wR_2 = 0.1324	R_1 = 0.0666, wR_2 = 0.1842
Final R indexes [all data]	R_1 = 0.1019, wR_2 = 0.2378	R_1 = 0.0581, wR_2 = 0.1404	R_1 = 0.0910, wR_2 = 0.2070
Largest diff. peak/hole/e Å^{-3}	0.49/-0.34	0.32/-0.31	0.78/-0.43
**CCDC No.**	**1831580**	**1831581**	**1831582**

For the THF solvate, the reflections could be indexed to two cells with similar figures of merit. Forced indexing to the smaller cell (a = 10.5354 Å, b = 11.1964 Å, c = 14.0341 Å, α = 79.285 °, β = 86.385 °, γ = 79.872 ° (V = 1600 Å3) with Z' = 1 and carrying out data reduction with this model resulted in disregarding several weak interstitial reflections. The subsequent model from this solution returned poor Uij values when ADPs were freely refined on several atoms, mainly involving the pyridine rings, and averaged two orientations of the lattice THF molecule. This model also displayed uniformly worse refinement statistics. By carrying out data reduction on the larger cell with Z' = 2, we obtained a much more reasonable model in which the two residues seem genuinely ordered with respect to the two orientations of the lattice THF molecule. This is by far the more chemically reasonable model, despite the two main residues exhibiting sufficiently similar geometries related by translational symmetry to flag automated warnings regarding possible missed symmetries.

Table S2. Summary of UV-vis absorption and emission spectral data for **TBNap** (1.0×10^{-6} M) in different solvents of varied polarity.

Solvent	Polarity Index (P')	Absorption maxima λ_{max} (nm)	Molar extinction coefficient ε (10^3) M^{-1} cm^{-1} [λ_{max} (nm)]	Fluorescence emission maxima λ_{max} (nm)	Stokes Shift (cm^{-1})
Toluene	2.4	385	13.9	470	4697.4
DCM	3.1	387	23.9	500	5839.8
THF	4.0	384	21.2	507	6317.8
ACN	5.8	385	17.3	534	7247.4
DMF	6.4	388	18.6	542	7323.0
DMSO	7.2	391	20.9	544	7193.1

Computational Details:

Time-dependent density functional theory (TD-DFT) calculations were performed with the CAM-B3LYP functional and 6-31G(d) basis set.[1] This function was chosen for its reliability in describing long-range charge-transfer excitations.[2] To account for solvent effects, the polarizable continuum model (PCM) was employed with tetrahydrofuran (THF) as the solvent, in agreement with experimental conditions. Vibrational frequency calculations confirmed that the optimized structures were local minima with no imaginary frequencies. Vertical excitation energies were obtained using time-dependent density functional theory (TD-DFT) at the same level of theory. For the absorption spectrum, the lowest 30 singlet excited states were calculated at the S_0 equilibrium geometry. Oscillator strengths and orbital contributions were examined, and in order to obtain the emission properties, the lowest singlet excited state (S_1) was optimized with TD-DFT (CAM-B3LYP/6-31G(d), PCM/THF), followed by vibrational frequency analysis, which confirmed the absence of negative frequencies. From this geometry, vertical emission energies were computed by calculating the transition back to S_0. From the optimized S_1 geometry, vertical emission energies were computed considering 10 singlet states, with the emission corresponding to the $S_1 \rightarrow S_0$ transition. The SCF calculations used tight convergence thresholds (SCF = VeryTight) and an ultrafine integration grid. All calculations were carried out using the Gaussian 16 program package (Revision C.01).[3]

References:

(1) Yanai, T.; Tew, D. P.; Handy, N. C. A New Hybrid Exchange–Correlation Functional Using the Coulomb-attenuating method (CAM-B3LYP), Chem. Phys. Lett., 2004, 393, 51−57.

(2) Gopikrishna, P.; Adil, L. R.; Iyer, P. K. Bridge-Driven Aggregation Control in Dibenzofulvene–naphthalimide based Donor Bridge–Acceptor Systems: Enabling Fluorescence Enhancement, Blue to Red Emission and Solvatochromism, Mater. Chem. Front., 2017, 1, 2590–2598.
(3) Frisch, M., *et al*. Gaussian 16, Revision C.01, Gaussian Inc., Wallingford, CT (2016).
